# Oligodendrocyte and Astrocyte Dynamics During Short‐Term Cuprizone Treatment

**DOI:** 10.1002/brb3.71660

**Published:** 2026-08-15

**Authors:** Lana Frankle, Mercy Oso, Amanda Riley, Riely Tomor, Davi Cecconi Checan, Hannah Lee, Kole Jarzembak, Sarah Sternbach, John Shelestak, Jennifer McDonough, Robert Clements

**Affiliations:** ^1^ School of Biomedical Sciences Kent State University Kent Ohio USA; ^2^ Department of Biological Sciences Kent State University Kent Ohio USA

**Keywords:** astrocytes, cuprizone, demyelination, oligodendrocytes

## Abstract

**Purpose:**

Glial cells, including oligodendrocytes (OLs), astrocytes, and microglia, are brain cells that support and dynamically interact with neurons and each other. These intercellular dynamics undergo changes during stress and disease states. Studies have reported cross talk between glial cells, such as astrocytes, microglia, and OL precursor cells (OPCs), and recruitment to central nervous system (CNS) lesions. However, the dynamics of this interaction are still unclear, especially during the early time point of exposure to cuprizone (CPZ).

**Methods:**

In this study, we exposed mice to CPZ treatment for a short duration (3 days and 1 week); we used immunohistochemistry to quantify Aspartoacylase (ASPA)‐positive OLs, complement component 3d (C3d)‐positive astrocytes (A1 subtype), and epithelial membrane protein 1  (Emp1)‐positive astrocytes (A2 subtype). Gene expression levels of myelinating OLs were quantified using reverse transcription polymerase chain reaction (RT‐PCR), and protein expression of C3d and Emp1 was quantified using Western blot.

**Finding:**

After 3 days of CPZ treatment, there was a significant increase in the expression of the astrocyte A2 marker (Emp1) and a significant decrease in mature OLs marked by ASPA. No significance was observed for C3d at 3 days and 1 week. Additionally, the Myelin Basic Protein (MBP) gene, which also shows the expression level of myelin, was significantly downregulated at 3 days. Previous work indicates that CPZ causes blood–brain barrier (BBB) permeability as early as 3 days, with subsequent microglial and astrocyte activation before demyelination.

**Conclusion:**

This study suggests an interaction between OLs death and astrocyte subtypes activation during CPZ treatment at early time points (3 days and 1 week) before overt demyelination. Understanding this cross talk and the changes in their activation and interaction is important for developing methods to prevent or reverse demyelination.

## Graphical abstract

Early cuprizone exposure (3 days to 1 week) results in the loss of mature oligodendrocytes and a shift in astrocyte subtypes before overt demyelination occurs. A1 astrocytes remained non‐significant; however, A2 astrocytes increased, indicating a protective, pro‐repair astrocyte–oligodendrocyte interaction that may be crucial for therapeutic intervention.

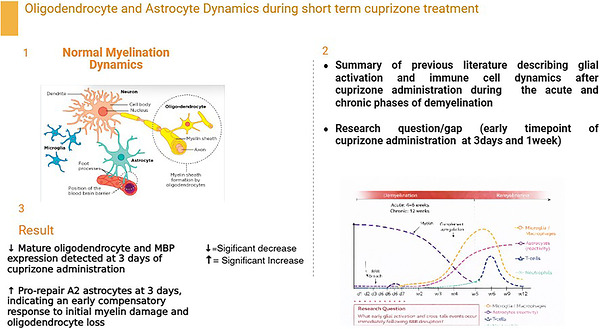

## 1 Introduction

Myelination is characterized by the acquisition of highly specialized myelin membrane around axons. This process is particularly active during the early years continuing into adolescence, and it plays a crucial role in the maturation and proper functioning of the nervous system through its contributions to learning and memory, as well as the overall efficiency of neural communication. During disease states such as multiple sclerosis (MS), Parkinson's disease (Xie et al. 2022), schizophrenia (Valdés‐Tovar et al. 2022), depression (Sacchet and Gotlib 2017), and Alzheimer's disease (Papuć and Rejdak 2018), demyelination is usually observed through damage caused to the protective cover (myelin sheath) that surrounds axons. Mature oligodendrocytes (OLs) are vital for forming myelin sheaths that wrap around axons. Concurrently, astrocytes and microglia release various molecules that regulate the development of OLs and myelination (Zhen‐Kun et al. 2022), and this process is repeated during disease when the myelin sheath is damaged and necessitates repair. Oligodendrocyte precursor cells (OPCs) respond by undergoing proliferation and differentiation to supplement the population of OLs. During this repair process, astrocytes and microglia enter an activated state, and microglia engulf fragments of the damaged myelin sheath (Zhen‐Kun et al. 2022). This intricate interplay between OPCs, OLs, astrocytes, and microglia highlights the dynamic nature of myelination and its repair mechanisms. Although OLs predominantly drive myelination, other glial cells contribute to this process, including astrocytes and microglia.

Most astrocyte research has focused on modulating neuronal function and synaptic transmission during disease. Still, little is known about the cross talk between astrocytes and OLs, which occurs via direct cell‐cell contact and by secreted cytokines, chemokines, exosomes, and signaling molecules. Additionally, this cross talk is essential for glial development, triggering disease onset and progression and stimulating regeneration and repair. Its critical role in homeostasis is most evident when this communication fails (Nutma et al. 2020).

Astrocytes cross talk with OLs can be beneficial or detrimental, especially the A1 subtype astrocyte reactivity, which stops cells from functioning to support and sustain neural connections and instead causes OL dysfunction and neuronal death following injury (Li et al. 2020). In contrast, another subtype of activated astrocytes called A2 astrocytes continues to promote neural health by upregulating neurotrophic genes that promote cell survival and anti‐inflammatory substances (Li et al. 2019). This type of activated astrocyte is marked by the upregulation of epithelial membrane protein 1 (Emp1) (Cunningham et al. 2019). Emp1 is involved in blood–brain barrier (BBB) tight junction formation and drug resistance (Abbott et al. 2010) in the cuprizone (CPZ) model, frequently used to model some aspects of MS, schizophrenia, and other diseases. CPZ toxicity acts differently in gray and white matter, with astrocytosis detected earlier in the cortex (after 2 weeks) than in the corpus callosum (after 5 weeks) (Wergeland et al. 2012). It is characterized by the loss of OLs rather than a direct attack on the myelin sheath (Kipp et al. 2009). This model can be used to study dynamic cellular cross talk and the interplay between glial cells during different states of demyelination and remyelination. In the CPZ model, the BBB is disrupted at 3 days (Shelestak et al. 2020), a time point that precedes demyelination and significant changes in the brain. In a mouse model of pericyte loss (another cell type of the BBB), in adulthood, myelin sheaths become thinner, white matter tracts become disorganized and vacuolized, and OLs undergo apoptosis. Pericytes stimulate OPCs differentiation after central nervous system (CNS) demyelination (de et al. 2017), and these findings suggest that pericyte loss can initiate OLs loss and affect subsequent regeneration by OPCs in white matter diseases.

It has been shown that CPZ‐induced OL death requires microglia recruitment and inflammatory cytokine release (Pasquini et al. 2007), and microglial activation may depend on astrocytic cytokine release (Clarner et al. 2015) following activation from astrocytes.

The influence of astrocytes on OL proliferation and differentiation is vital for the remyelination potential of white matter lesions. Despite this, the precise mechanisms through which astrocytes impact remyelination are still unclear.

Before now, acute demyelination and loss of OLs have been reported at 4–6 weeks, and astrocyte and microglial activation at 2 weeks and 1 week, respectively. This study suggests a relationship between the death of OLs and activation of Emp1 at an early time point (3 days) of CPZ administration, which might be a window to explore therapies before structural damage occurs. For a long time, OLs were thought to be the victim in this cascade, but emerging evidence shows that they might be actively involved in the initiation. Therefore, this study aims to understand the dynamics of OLs and the activation of astrocytes (A1 and A2 markers) during CPZ treatment at early time points. Understanding this cross talk and the change in their interactions is essential for developing therapies to prevent or reverse demyelination.

## 2 Materials and Methods

### 2.1 Animals and Diet

A total of 48 male C57BL/6J mice were obtained from Jackson Laboratory and used in this study. Mice were assigned to either the CPZ treatment group or the control group at two experimental time points (3 days and 1 week). For each time point, 12 mice were fed a diet containing 0.3% of CPZ chow (Envigo), while the other 12 mice in the control group were fed regular chow. Within each group, six mice were designated for RNA and protein analyses, and the remaining six mice were used for immunofluorescence studies. CPZ‐containing chow was replaced every 2 days to maintain its potency, as CPZ is susceptible to degradation at room temperature. To ensure consistent CPZ intake, mice were housed individually in wire‐bottom cages to prevent food hoarding and consumption of older, less potent CPZ pellets.

Animals were weighed every 2 days because weight loss is a well‐known effect of CPZ treatment, and changes in body weight are thus an indicator of the toxin's effectiveness. In addition, any animal that lost > 20% of its body weight was removed from the study following the experimental protocol accepted by the Institutional Animal Care and Use Committee (IACUC) (Protocol number #557 RC 23‐13).

### 2.2 Tissue Processing

At the end of each time point, mice were euthanized by cervical dislocation, and brains were dissected. For molecular analyses, brain tissue was collected and immediately frozen for subsequent RNA and protein extraction. For immunofluorescence studies, brains were fixed in 4% paraformaldehyde (PFA), cryoprotected, and sectioned at a thickness of 100 µm.

### 2.3 Protein Extraction

Brains frozen at −80°C were separated into white matter and gray matter using a razor. Each sample was homogenized on ice using a 2‐mL glass mechanical homogenizer (Cole‐Parmer) in 150 µL premade RIPA radioimmunoprecipitation assay (RIPA) lysis buffer (Thermofisher) with 1% Protease Inhibitor Cocktail (Abcam) added by volume to prevent protein degradation. The tubes were incubated on ice, rotated at 4°C, and spun on a microcentrifuge (Brinkmann Centrifuge 5415). The supernatant was then collected as the cytoplasmic fraction, frozen at −20°C.

### 2.4 RNA Extraction

Note that 50–100 mg of the frozen brain sample at −80°C was used for RNA extraction. Each sample was homogenized on ice using a 2‐mL glass mechanical homogenizer (Cole‐Parmer) using the Trizol reagent protocol for RNA extraction. The extracted RNA was diluted to 100 ng in 1 µL across all prepared reactions.

### 2.5 Western Blot Protein Expression Measurement

Western blots assessed corpus callosum and cortical levels of C3d and Emp1. A protein concentration assay was run on all samples using a BIORAD protein assay kit and SpectraMax 4000 plate reader with 595 wavelength absorbance. The standard curve was set using Bovine Serum Albumin (BSA) dilutions as protein standards. Protein was denatured with 1:1 Laemmli loading buffer (BIO‐RAD) by boiling for 10 min on a dry bath (Thermolyne Dri‐Bath) at 100°C and spun down on a microcentrifuge (Eppendorf Centrifuge 5414) for 10 s before being loaded into 15‐well 1 mm 10% gels (BIO‐RAD) with a total volume of 10–15 uL per lane and a total protein level of 15 ug per lane, with 3 µL PageRuler 10–250 kd Prestained Protein Ladder (Thermofisher). Gels were run at 120 V using a BIORAD electrophoresis rig until total ladder separation was achieved. Protein was transferred to nitrocellulose or PVDF membrane (BIO‐RAD) at 55 V for 1 h. Blots were blocked using 5% BSA for 45 min to limit background staining, sealed in plastic using a heat sealer (Impulse Sealer), and incubated overnight at 4°C with 1:1000 primary antibody in BSA at 4°C. Primary antibodies were C3d (goat host, R&D Systems AF‐2655) and Emp1 (rabbit host, Abcam, ab202975). Blots were washed in Tris‐buffered saline with Tween‐20 (TBST) and incubated for 40 min at room temperature in 1:10,000 secondary antibodies in BSA according to the primary host. (Donkey anti‐goat or donkey anti‐rabbit, Santa Cruz.) GAPDH (Invitrogen) or Vinculin (Abcam) was a protein loading control. Blots were imaged using luminol (ImmunoCruz, Santa Cruz Biotechnology Inc.) on ImageQuant LAS 4000 at high or super‐resolution. The resulting images were saved as 16‐bit TIF files, and band brightness was assessed using the image processing software FIJI image analysis function for gels.

### 2.6 RT‐PCR

This study analyzed gene expression levels of OPCs and myelinating OLs. Agilent Brilliant III Ultra‐Fast SYBR Green qRT‐PCR Master Mix was used for the RT‐PCR following the manufacturer's protocol. The ΔΔCt technique was used to measure relative gene expression levels of Platelet‐Derived Growth Factor Receptor Alpha (PDGFR), Myelin Oligodendrocyte Glycoprotein (MOG), Myelin Associated Glycoprotein (MAG), and Myelin Basic Protein (MBP). Gene‐specific primers were sourced from Integrated DNA Technologies (IDT).

The main objective was to determine whether these target genes changed expression patterns (upregulation or downregulation) at various points in the experimental timeline. The standard housekeeping gene, β‐actin (Actb), was consistently expressed across all experimental groups (Table 1).

**TABLE 1:** Primers sequence sourced from Integrated DNA Technologies (IDT).

Primers	Sequence
MOG FW	ATATCTGGCAAGGGTGACGTG
MOG RV	GCCCAACCTCCATGTAAGCA
MAG FW	GGAGCCCAAGGGACTGTAAG
MAG RV	AGTGGCCTTTCAACCAAGTCT
PDFGRA FW	GTAGTCCCCACCCCCACTC
PDGFRA RV	TAGCTCCTGAGACCTGCACC
Reference gene (Beta‐actin)	GACTCATCGTACTCCTGCTTG
Reference gene (Beta‐actin)	GATTACTGCTCTGGCTCCTAG
MBP FW	TCCATCGGGCGCTTCTTTAG
MBP RV	CCTTGTACATGTGGCACAGC

Abbreviations: FW, forward; MAG, Myelin Associated Glycoprotein; MBP, Myelin Basic Protein; MOG, Myelin Oligodendrocyte Glycoprotein; PDGFR, Platelet‐Derived Growth Factor Receptor Alpha; RV, reverse.

### 2.7 Immunohistochemistry

Sections were incubated for 24 h at room temperature and blocked with 3% donkey serum, 0.5% Triton‐X, and primary antibodies for C3d (R&D Systems AF‐2655) at 1:40, Emp1 at 1:100 (rabbit host, Abcam, ab202975), and Aspartoacylase (ASPA, 2 µg/mL, 1:500, EMD Millipore, rabbit ABN1698), in PBS. After primary staining, slices were washed 3× in PBS and then incubated with secondary antibodies C3d (Invitrogen 488 anti‐goat) or Emp1 (Invitrogen 488 anti‐rabbit) for 24 h and ASPA (1:400, species‐specific Alexa 488) for 6 h followed by 1:2000 DAPI (Invitrogen). Isometric 3D image stacks were taken at 20X using an Olympus Fluoview 1000X confocal microscope.

### 2.8 Statistics

Statistical analysis of densitometry data was performed, and results were presented as mean and standard error. Significance was assessed at 0.05 using a two‐tailed *t*‐test. Analysis of the result images after mask subtraction for Emp1 and C3d, and cell counts for OLs, was also performed. Significance thresholds were calculated using a two‐tailed Student's *t*‐test in GraphPad Prism, and data are presented as mean ± SEM. *p ≤ 0.05, **p ≤ 0.01, and ***p ≤ 0.001.

## 3 Results

**FIGURE 1:**
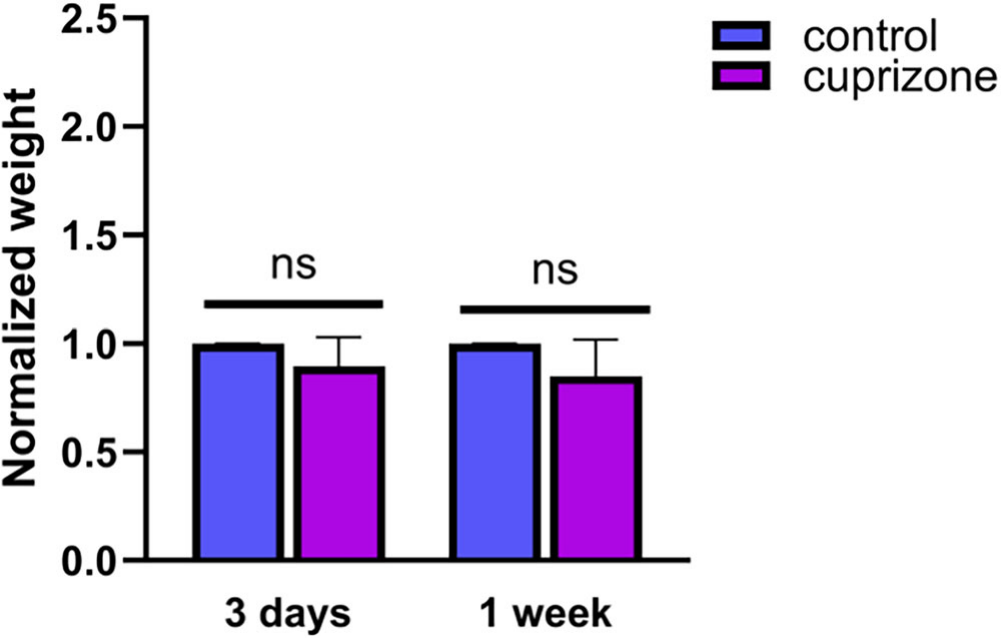
Body weight analysis. Body weight was measured in cuprizone (CPZ)‐treated mice and control mice at each time point throughout the study. Statistical significance was determined using a two‐tailed Student's *t*‐test. No significant differences (ns) were observed between the groups at 3 days and 1 week of treatment. Data are presented as mean ± SEM.

Mice treated with CPZ at both experimental time points did not exhibit any overt signs of physical disability during the study. However, CPZ‐treated mice experience body weight loss, consistent with previous reports using the CPZ model (Praet et al. 2014). Their weight was lower than that of the control group at every time point. The control mice slightly gained weight, consistent with juveniles being fed regular chow ad libitum (Figure 1). Although there was a slight increase in weight for control mice, it was not significant when compared with the CPZ mice.

**FIGURE 2:**
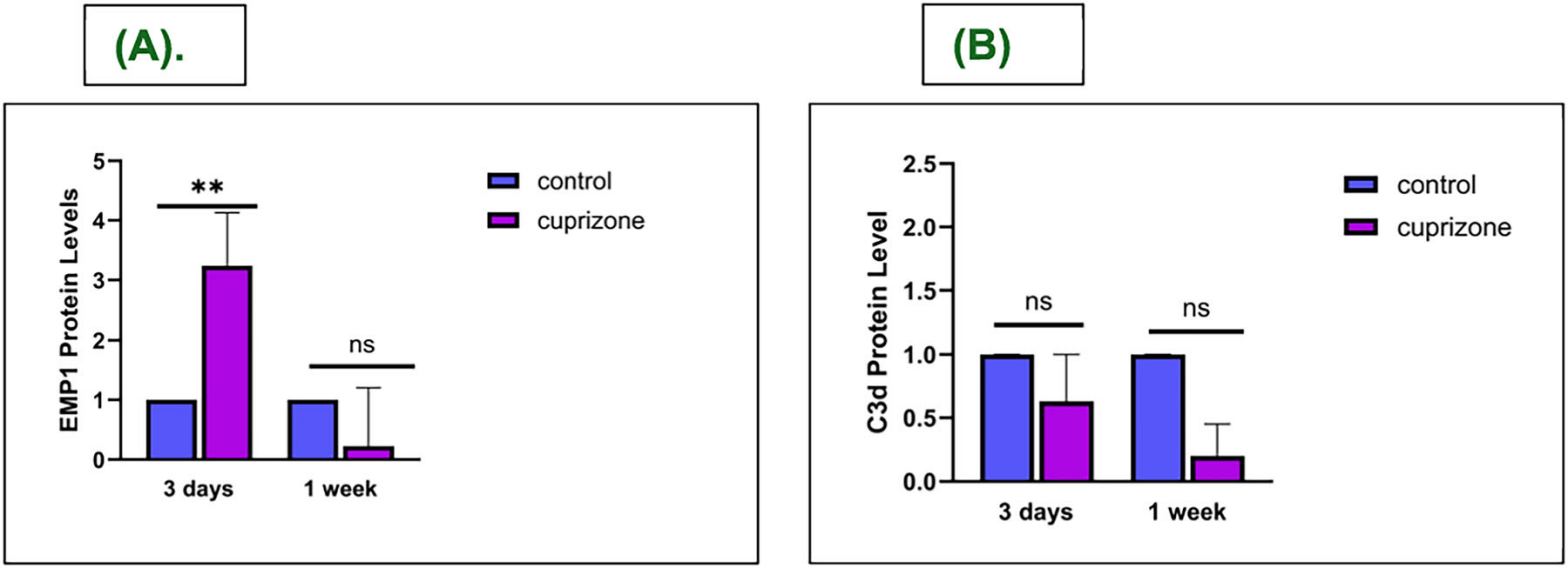
Quantification of epithelial membrane protein 1 (Emp1) and complement component 3d (C3d) protein expression in the corpus callosum. Densitometric analysis of Emp1 and C3d protein levels in the corpus callosum of control and cuprizone (CPZ)‐treated mice. (A) Emp1 protein expression was significantly increased in CPZ‐treated mice (*n* = 6) compared with control mice (*n* = 6) after 3 days of treatment. In contrast, (B) C3d protein expression did not differ significantly between CPZ‐treated mice (*n* = 6) and control mice (*n* = 6) at any time point within the experimental time points. Data are presented as mean ± SEM. **p ≤ 0.01 versus control; ns = not significant, as determined by a two‐tailed Student's *t*‐test.

After 3 days and 1 week of CPZ exposure, brain tissue was subjected to Western blot and confocal microscopy imaging to assess changes in astrocyte activation after CPZ administration. At 3 days, CPZ caused a significant increase in Emp1 protein levels compared with control, but at 1 week, no significant difference was observed in the corpus callosum. For C3d, no significant difference was detected at the protein level in the corpus callosum at both 3 days and 1 week (Figure 2).

**FIGURE 3:**
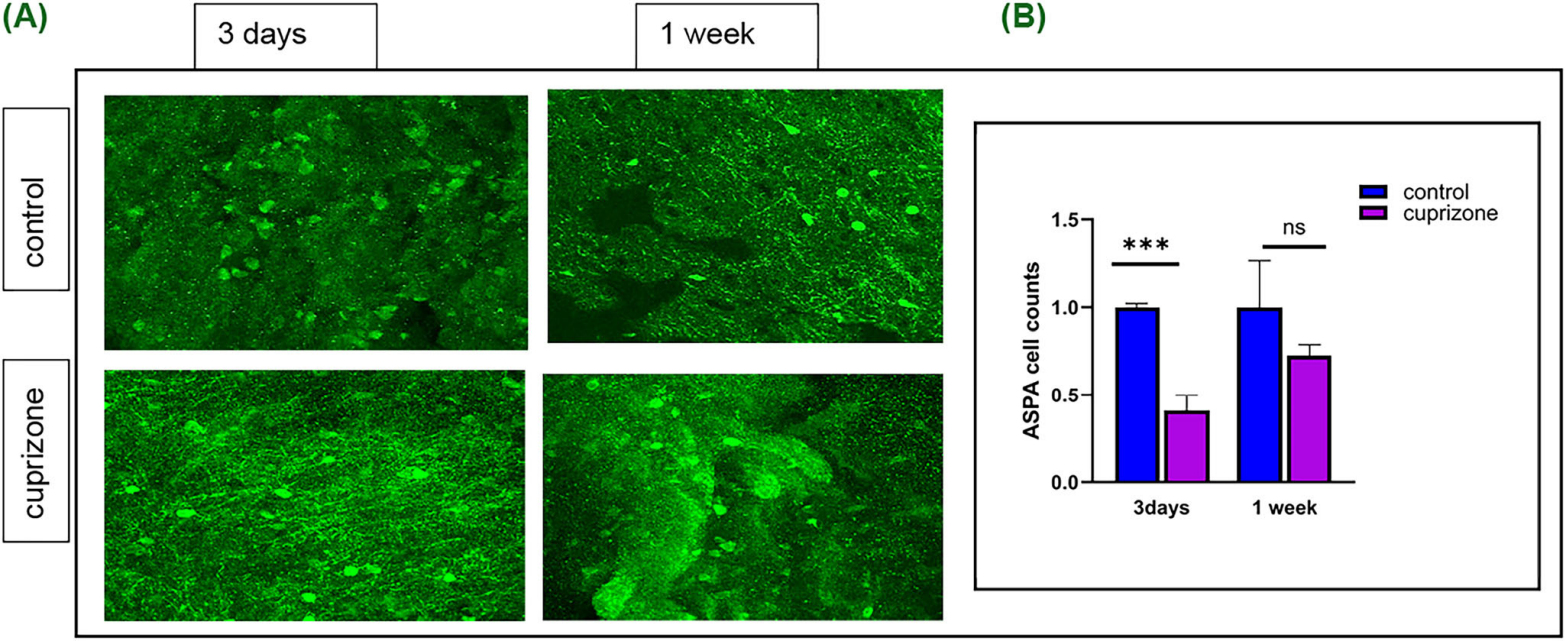
Reduction in aspartoacylase (ASPA)‐positive oligodendrocytes following cuprizone (CPZ) administration. (A) Representative immunofluorescence images of ASPA‐positive oligodendrocytes in the corpus callosum of control and CPZ‐treated mice after 3 days and 1 week of treatment. (B) Quantification of ASPA‐positive cells is shown. Male mice (*n* = 6 per group) were analyzed at each time point. CPZ treatment resulted in a significant reduction in the number of ASPA‐positive cells at 3 days compared with controls. Data are presented as mean ± SEM. ***p ≤ 0.001 versus control; ns = not significant, as determined by a two‐tailed Student's *t*‐test. Scale bar = 16 µm; magnification = 20×.

Immunofluorescence staining for ASPA in the corpus callosum of the brain at 3 days and 1 week after CPZ administration was performed to determine the cell count of OLs during CPZ treatment. At 3 days, there was a significant decrease in the number of ASPA cells compared to that of the control animal, while at 1 week, there was no significance, but the number of ASPA cells seemed to be elevated (Figure 3b).

**FIGURE 4:**
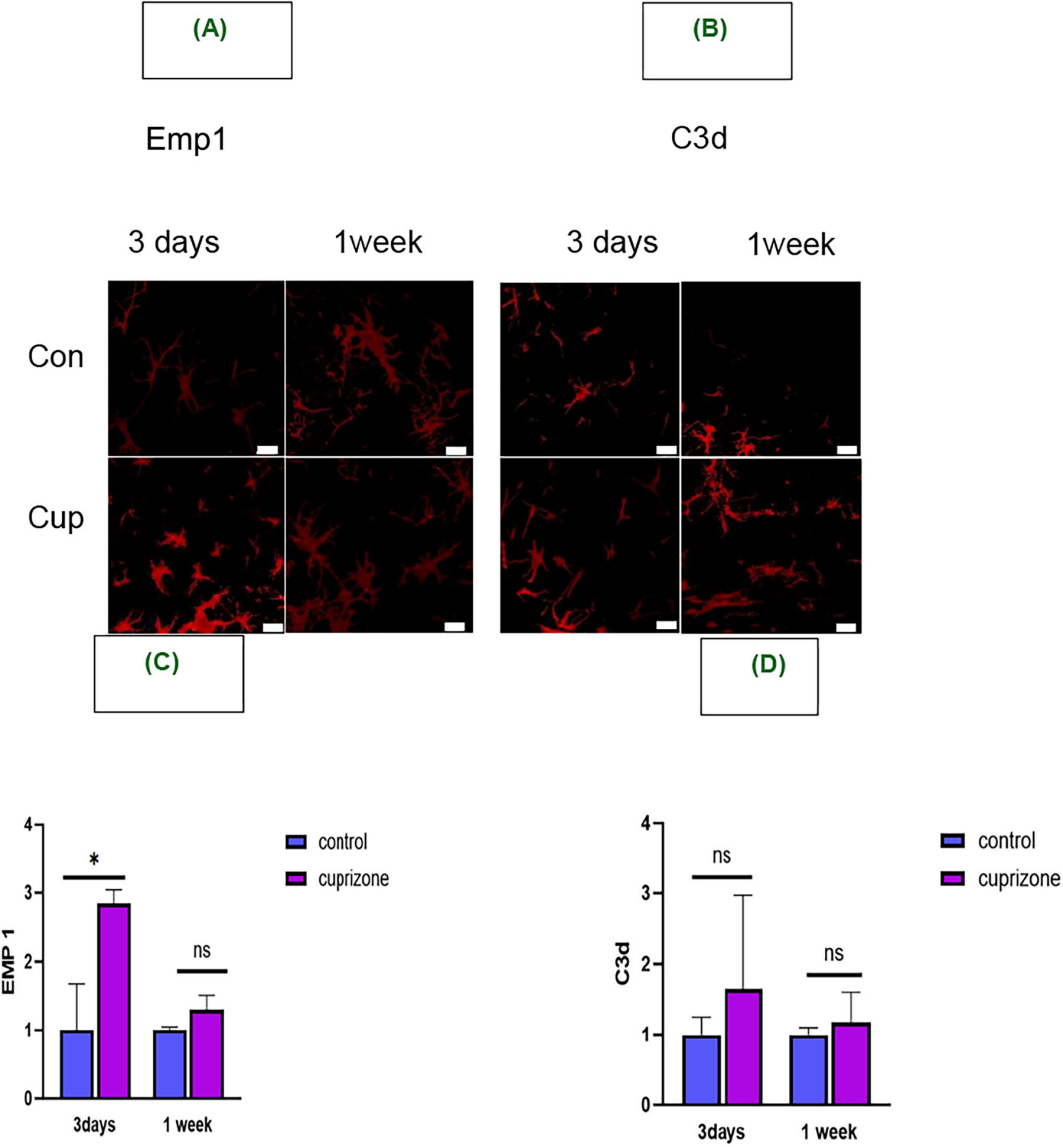
Immunofluorescence image of (A) epithelial membrane protein 1 (Emp1) and (B) complement component 3d (C3d) in the corpus callosum following cuprizone (CPZ) treatment. Immunofluorescence analysis of (C) Emp1 and (D) C3d astrocyte subtypes revealed significant changes in Emp1 brightness in the corpus callosum at 3 days in the mice treated with CPZ compared to controls, but not at 1 week, as shown in the bar graph. Astrocytes were stained with glial fibrillary acidic protein (GFAP), and a binary mask was created, which was then used to crop the Emp1 or C3d channel; the average intensity of the resulting image was used to quantify the presence of Emp1/C3d in labeled astrocytes only. No significant differences were observed in C3d fluorescence at any time point. Male mice (*n* = 6) per group were used at different time points. Scale bar 10 µm and magnification: 20x. Data are presented as mean ± SEM. *p ≤ 0.05 compared with control; ns = not significant, using a two‐tailed Student's *t*‐test.

Immunofluorescence staining for C3d and Emp1 was also performed to determine the subtypes of astrocytes present. To quantify the amount of A1 or A2 markers in astrocytes, brain tissue was double‐stained for Emp1 or C3d and Glial Fibrillary Acidic Protein (GFAP), and the degree of overlapping intensity within the GFAP channel was analyzed at different experimental time points. C3d, the A1 subtype of astrocytes, was not significantly different in the corpus callosum at either 3 days or 1 week. However, Emp1, which is a measure of the A2 subtype of astrocytes, was significantly different at 3 days but not at 1 week in the corpus callosum (Figure 4).

**FIGURE 5:**
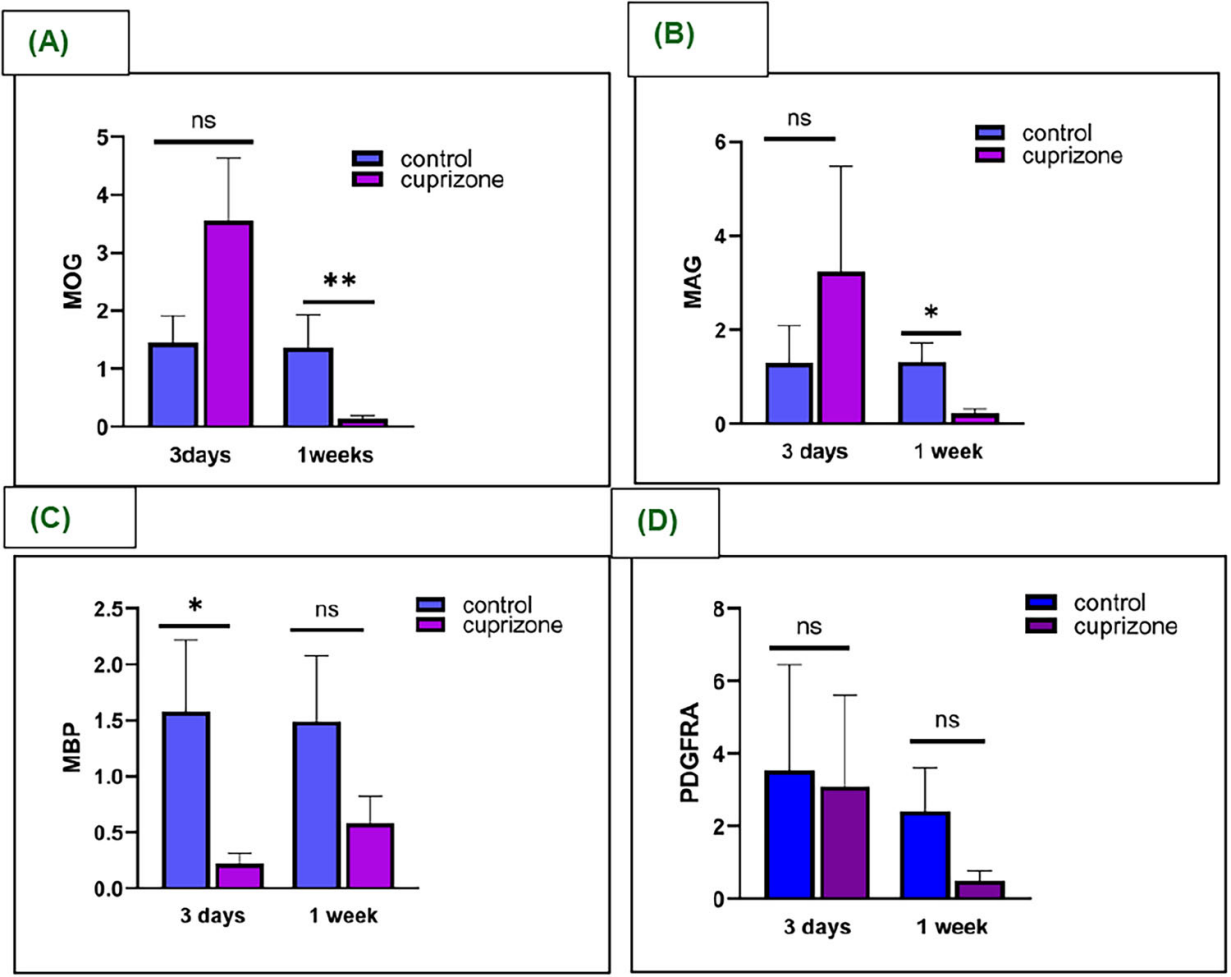
Gene expression profile of myelinating oligodendrocytes and oligodendrocyte precursor cells using reverse transcription polymerase chain reaction. Gene expression levels of (A) Myelin Oligodendrocyte Glycoprotein (MOG), (B) Myelin‐Associated Glycoprotein (MAG), (C) Myelin Basic Protein (MBP), and (D) Platelet‐Derived Growth Factor Receptor Alpha (PDGFRA) were evaluated in the corpus callosum of male mice (*n* = 6 per group) at different time points. A significant downregulation of MOG and MAG was observed at 1 week (A and B), while MBP was significantly downregulated at 3 days (C). Data are presented as mean ± SEM. *p ≤ 0.05, **p < 0.01 versus control; ns = not significant, as determined by a two‐tailed Student's *t*‐test.

To further understand the dynamics of OLs during CPZ treatment, we examined the gene expression levels of myelinating OLs and OPCs at different time points (3 days and 1 week). RNA was extracted and converted to cDNA using RT‐PCR to analyze the upregulation and downregulation of MOG, MAG, MBP, and PDGFRA genes in the CPZ‐treated brain compared with those in the control brain. Significant downregulation of MOG and MAG was observed at 1 week (Figure 5a,b), MBP was downregulated at 3 days (Figure 5c), and no significant difference was observed for PDGRFA at 3 days and 1 week (Figure 5d).

## 4 Discussion

Previous studies in our lab have shown that mast cell activation occurs alongside a decrease in occludin expression, a tight junction protein involved in maintaining BBB integrity, in the corpus callosum as early as 3 days. This is followed by microglial activation observed after 1 week. These findings highlight the importance of investigating astrocyte and OL activity during these early time points, as they may reveal critical functional changes preceding the structural changes associated with demyelination. Additionally, most studies have focused primarily on demyelination itself, with limited attention given to the events preceding its onset. Exploring these early time points is essential, as they could provide insights into mechanisms and proper timing to halt or delay the onset of demyelination.

This study was conducted on the corpus callosum of mice brain‐fed a diet containing 0.3% CPZ to investigate its effects on astrocyte and OL dynamics.

The study showed a significant increase in the astrocyte reactive marker A2 level. A2 Astrocyte marker Emp1 in the corpus callosum was significantly elevated at 3 days but not at 1 week (Figure 2). This pattern could represent a countering trend in the levels of A2‐activated astrocytes and endothelial cell integrity in the BBB to OL death, or it could describe shifting patterns in A2 astrocyte activation. Additionally, the colocalized brightness measurements for Emp1 in the corpus callosum mirrored the time points at which the protein levels increased at 3 days (Figure 4c).

Previous studies revealed that mast cell activation can be measured after 3 days and that microglial activation can be measured after 1 week (Shelestak et al. 2020). This same study also showed a decrease in occludin expression, a tight junction protein involved in BBB integrity, in the corpus callosum at 3 days. This finding is relevant when considering the levels of Emp1, which, in addition to being a marker for A2 astrocytes, is also expressed by endothelial cells and the levels of which in this study are elevated in the corpus callosum at 3 days. Whether this increase results as a compensatory mechanism for the decrease in occludin or merely represents an increase in A2 astrocytes that outweighs or is independent of endothelial cell Emp1 could be investigated in subsequent studies. Also, previous studies induced with the myelin toxin lysolecithin (LPC) have shown that the transplantation of A2 astrocytes into the lesion site of the spinal cord in mice significantly improved motor function recovery, reduced glial scar formation, and enhanced neurofilament formation at the injury site compared to the transplantation of A1 astrocytes. Additionally, Luxol fast blue staining and electron microscopy revealed better preservation of myelin with a more organized structure in the A2‐astrocyte group than in the A1‐transplanted group. These findings suggest that A2 astrocytes offer superior support for remyelination and structural integrity at the site of spinal cord injury (Molina‐Gonzalez et al. 2023).

It is known that A2 releases numerous neuroprotective cytokines and regulates brain homeostasis (Li et al. 2019). The activation of A2 astrocytes during CPZ treatment after 3 days suggests a compensatory response to help counter the leaky BBB and possibly the loss of OLs. This cross talk suggests that the BBB, OLs death, and the A2 astrocyte activation might co‐occur during CPZ administration. This study cannot say which event occurred first; therefore, early time points such as 1 and 2 days can be examined, although a study reported a 65% loss of OLs at 2 days (Jhelum et al. 2020), which may also suggest that OL death might initiate the whole cascade. Taken together, these findings indicate that the cross talk seems to occur concurrently, and this was reflected at 1 week when there was an increase in OLs, possibly because of the activation of A2‐like astrocytes since findings have suggested that if A1‐like astrocytes return to the A2‐like type, OL lineage cells can mature and protect against the progression of white matter injury (Chang et al. 2023), which is similar to the finding that A2 astrocytes increase the preservation of myelin with organized structures when transplanted into the lesion site of the spinal cord rather than A1 astrocytes (Chang et al. 2023).

For C3d, a marker for A1 astrocytes known to release cytotoxic chemicals that cause neurons and OLs to die, there was no protein increase at any time during the study in the corpus callosum. This may suggest that the activation of C3d at the corpus callosum does not occur during this early period of exposure to CPZ but occurs later during demyelination. Although other studies have reported the activation of cortical C3d in CPZ treatment at 1 week and subsequent decrease, which shows a dynamic pattern of C3d activation at different time points (Wergeland et al. 2012), no changes were detected in the corpus callosum in this study. While C3d can be expressed by multiple cell types, including astrocytes, microglia, mast cells, and macrophages, its interpretation in this study was refined by co‐localization with GFAP to specifically identify its expression in astrocytes. C3d remains a robust marker of inflammatory and immune activation, regardless of cellular source. The timing of its expression reflects a defined stage of immune response, which may represent a critical therapeutic window for intervention in disease conditions such as MS.

The CPZ neurotoxicity animal model is widely used in some aspects of MS research, and it involves the administration of the toxin CPZ to induce OL apoptosis. Upon its administration, mice typically exhibit profound demyelination in the corpus callosum, and OL apoptosis is observed, together with the recruitment of microglia/macrophages, astrocytes, and OPCs (Matsushima and Morell 2001).

Most previous studies were carried out between 3 and 6 weeks. We looked at earlier times, 3 days and 1 week. We observed a significant decrease in OLs at 3 days and a subsequent increase at 1 week, similar to the result of the gene expression of MBP. This result suggests functional damage occurred earlier than the overt demyelination experienced later. This may indicate that the death of OLs may be a primary effect of CPZ and not due to other cells' activities at this time point. More research should be done to verify this claim. We may also suggest that the direct effect of CPZ on OLs can initiate a chain of reactions that activate other cells and subsequently indirectly cause more death of OLs, which is in line with a recent review article that suggests that OLs might be involved in its downfall during CPZ treatment (Zirngibl et al. 2022).

Our gene expression data revealed no downregulation of the expression of myelin‐associated genes such as MAG and MOG at 3 days. These two proteins are crucial for myelin formation, maintenance, and stability. This result is supported by a previous report, where no demyelination was observed at 3 days of CPZ treatment using fluoromyelin (Shelestak et al. 2020), but at 1 week, there was a significant downregulation of the genes, suggesting that myelin integrity may be affected as early as 1 week. This result is supported by another finding, showing that CPZ consumption may induce demyelination by reducing myelin protein and lipid synthesis, thereby destabilizing the myelin sheath. Morell and colleagues reported that the messenger ribonucleic acid (mRNA) levels of various myelin proteins, such as MAG and MBP, decreased by 75% and 50%, respectively, in the first week after the CPZ diet in rats (Morell et al. 1998). However, they also suggested that the lower synthesis may be a consequence rather than a driver of OL toxicity.

The expression of PDFRA, a marker of OPCs, was downregulated at 1 week. However, the difference was not significant, which may suggest that OPCs differentiate into OLs as early as 1 week, and these cells can play a compensatory role. This indicates that at 1 week, the OPCs can still differentiate to remyelinate the axons. This is not the case after prolonged exposure to CPZ. This is also observed in MS during the relapsing‐remitting phase when the individual seems to improve for a while.

## 5 Conclusion

In this study, we investigated the dynamics of cell interaction and cross talk between astrocytes and OLs at early time point of CPZ administration, and we observed a non‐linear interaction. We suggest that understanding this very early cascade that eventually leads to demyelination after a period might help target cellular cross talk, specifically by trying to manipulate this cascade to develop therapies designed to target specific disease phases.

## Author Contributions

**Lana Frankle**: conceptualization, investigation, methodology, writing – original draft, writing – review and editing, software, formal analysis, data curation, validation, visualization, project administration. **Mercy Oso**: conceptualization, investigation, methodology, writing – original draft, writing – review and editing, software, formal analysis, data curation, validation, visualization, project administration. **Amanda Riley**: investigation. **Riely Tomor**: software. **Davi Cecconi Checan**: software. **Hannah Lee**: investigation, visualization. **Kole Jarzembak**: investigation. **Sarah Sternbach**: software, formal analysis. **John Shelestak**: formal analysis. **Jennifer McDonough**: methodology, validation, writing – review and editing. **Robert Clements**: conceptualization, funding acquisition, formal analysis, software, validation, resources, project administration, supervision, writing – review and editing. All authors reviewed and approved the final manuscript.

## Funding

National Institutes of Health supported this project with project number 1R15GM139115‐01.

## Ethics Statement

The Kent State University Institutional Animal Care and Use Committee (IACUC) accepted and approved the experimental protocol. Protocol number #557 RC 23‐13.

## Conflicts of Interest

The authors declare no conflicts of interest.

## Data Availability

The datasets used and analyzed during the current study are available from the corresponding author on request.
